# Relationship of POLST to Hospitalization and ICU Care Among Nursing Home Residents in California

**DOI:** 10.1007/s11606-023-08357-3

**Published:** 2023-08-24

**Authors:** David Zingmond, David Powell, Lee A. Jennings, Jose J. Escarce, Li-Jung Liang, Punam Parikh, Neil S. Wenger

**Affiliations:** 1 Division of General Internal Medicine and Health Services Research, David Geffen School of Medicine, University of California, Los Angeles, Los Angeles, CA USA; 2 RAND Health, Santa Monica, CA USA; 3 Reynolds Section of Geriatrics, Department of Medicine, University of Oklahoma Health Sciences Center, Oklahoma City, USA

**Keywords:** POLST, health care utilization, end-of-life care, nursing home, long-term care.

## Abstract

**Background:**

Physician Orders for Life Sustaining Treatment (POLST) document instructions for intensity of care based upon patient care preferences. POLST forms generally reflect patients’ wishes and dictate subsequent medical care, but it is not known how POLST use and content among nursing home residents is associated with inpatient utilization across a large population.

**Objective:**

Evaluate the relationship between POLST use and content with hospital utilization among nursing home residents in California.

**Design:**

Retrospective cohort study using the Minimum Data Set linked to California Section S (POLST documentation), the Medicare Beneficiary Summary File, and Medicare line item claims.

**Patients:**

California nursing home residents with Medicare fee-for-service insurance, 2011–2016.

**Main Measures:**

Hospitalization, days in the hospital, and days in the intensive care unit (ICU) after adjustment for resident and nursing home characteristics.

**Key Results:**

The 1,112,834 residents had a completed and signed (valid) POLST containing orders for CPR with Full treatment 29.6% of resident-time (in person-years) and a DNR order with Selective treatment or Comfort care 27.1% of resident-time. Unsigned POLSTs accounted for 11.3% of resident-time. Residents experienced 14 hospitalizations and a mean of 120 hospital days and 37 ICU days per 100 person-years. Residents with a POLST indicating CPR Full treatment had utilization nearly identical to residents without a POLST. A gradient of decreased utilization was related to lower intensity of care orders. Compared to residents without a POLST, residents with a POLST indicating DNR Comfort care spent 56 fewer days in the hospital and 22 fewer days in the ICU per 100 person-years. Unsigned POLST had a weaker and less consistent relationship with hospital utilization.

**Conclusions:**

Among California NH residents, there is a direct relationship between intensity of care preferences in POLST and hospital utilization. These findings emphasize the importance of a valid POLST capturing informed preferences for nursing home residents.

## INTRODUCTION

As patients become frail or lose their ability to express their wishes, capturing their preferences to guide medical care becomes a priority. Physician Orders for Life Sustaining Treatment (POLST) is a medical legal document that contains care intensity directions (resuscitation, selective treatments and comfort care, and medically assisted nutrition) that may complement an advance directive by converting an individual’s wishes regarding life-sustaining treatment and resuscitation into physician orders.

The effect of eliciting care intensity preferences on health care utilization is not well understood in chronically ill populations, such as nursing home (NH) residents. Prior work shows that care preferences—when known—are generally followed.^1,2^ A systematic review of POLST use found consistent evidence that treatment limitations recorded in a POLST were associated with less in-hospital death and less high-intensity treatment at the end of life.^3^ Studies of POLST among selected samples of NH residents show that orders prescribing less aggressive treatment are associated with less hospitalization and less hospital-based intensive treatment.^4–6^ However, the impact of POLST on hospital utilization has not been examined in population-based studies of NH residents.

We endeavored to understand the relationship between having a POLST and POLST content with hospital utilization accounting for resident characteristics across Medicare fee-for-service enrollees in California NHs from 2011 to 2016. We hypothesized that having a POLST containing instructions for lower intensity treatment would be associated with less utilization as measured by hospitalization, days in the hospital, and days in an intensive care unit (ICU).

## METHODS

We examined the relationship between POLST and utilization using the Minimum Data Set (MDS) linked to the California MDS Section S, Medicare Beneficiary Summary File (MBSF), Medicare Provider Analysis and Review (MedPAR) file, and Medicare line item claims. We obtained the California MDS 2011 to 2016 from the Centers for Medicare and Medicaid Services and the California Section S from the California Department of Public Health, Center for Health Care Quality. The MDS is a standardized, federally mandated health status assessment tool completed for all residents in a Medicare- and/or Medicaid-certified long-term care facility. MDS collects detailed demographic and clinical information on admission to the NH, quarterly, at a change in clinical status, and at discharge or death. MDS captures patient health status, cognitive status, and functional status.

The supplemental Section S containing questions about the use of POLST^7^ was added to the California MDS in 2010. Section S reports whether or not a POLST is in the chart and captures content from the most recently completed POLST, including Part A (CPR/DNR preferences), Part B (preferences regarding medical interventions), and Part C (artificially administered nutrition). It includes whether the patient (or a legally recognized decision-maker) and physician signed the POLST, but does not indicate date of completion. Changes in POLST occur asynchronously with MDS assessments and are captured at the next MDS assessment. Approximately 1% of MDS records with generally required POLST reporting (comprehensive, quarterly, and tracking assessments) had no Section S records, most likely associated with a nursing home that is exempted from completing the California Section S (accepting only private pay residents). The California MDS Section S is a unique record of POLST documentation. Two other states (West Virginia and Louisiana) report on their respective POLST forms, but neither state is as large or as diverse as California.

We created a longitudinal description of POLST completion and intensity of care preferences from 2011 to 2016, constituting the primary predictor for these analyses across MDS assessment segments. Each segment was weighted by duration of segment and normalized to patient-year (viz. “resident-time”). We classified responses as valid (Part A completed and both signatures present); signature missing (Part A completed and one or both signatures absent); POLST absent or missing Part A; and missing Section S. Among valid and signature-missing POLSTs, we categorized intensity of care preferences—we categorized resuscitation (CPR versus DNR) and further categorized patients within these groupings according to the desired level of care chosen in Part B—Full treatment, Selected treatment, and Comfort-focused treatment. We extrapolated POLST information in four circumstances in which POLST was not reliably captured in MDS assessment.^8^

Patient characteristics were derived from MDS assessments, including age, race/ethnicity, gender, cognitive status, functional status, comorbidity, and long-stay status. Cognitive status was measured using the Cognitive Function Scale.^9^ Functional status was measured using the Activities of Daily Living (ADL) Scale.^1^ We created a Charlson Comorbidity Index based upon conditions reported in MDS.^10^ Patients were defined as long-stay if they had greater than 100 days in a NH within a year (possibly non-consecutive).^11^ A resident defined as long-stay remained long-stay for the duration of the study.

Utilization measures were derived from the MBSF and MedPAR records. For each patient, we tallied hospitalizations, days in the hospital, and ICU days. Each of these measures was summed within an MDS record segment (viz. the period of time following an MDS assessment until the day before the next MDS assessment). For the last MDS assessment, we truncated the observation period to 90 days. For extended gaps between MDS assessments (corresponding to a patient leaving a NH), we limited the MDS segment to 90 days.

In multivariable regression models, we estimated the conditional relationship of POLST preferences and utilization (number of hospitalizations, hospital days, and ICU days). We divided each resident’s year into “segments” (defined by days between assessments), and we studied the count of each outcome per segment scaled by the length (in days) of that segment. Using linear regression, we studied the relationship between these scaled outcomes with whether the patient had a valid POLST and the content of the POLST. Estimates relative to the excluded category of not having a POLST (or Section A incomplete) and accounted for resident fixed effects, resident-varying characteristics (age, cognitive status, functional status, comorbidity index score, and whether ADL was imputed), and NH fixed effects. We estimated health care utilization using multiple linear regression weighted by the number of days in the segment. Standard errors were adjusted for individual-level clustering. Results were normalized to annualized measures by multiplying by 365. Because of the large sample size and multiple comparisons, we considered *p* < 0.001 as our cut-off for statistical significance.

In sensitivity analyses, we adjusted the cut-offs for the definition of long-stay, refined the number of days for truncation of end assessments, and excluded individuals without comprehensive or quarterly MDS assessments. None of the sensitivity analyses substantially changed the results.

This study was approved by the UCLA Office for the Protection of Research Subjects (IRB# 17-001534), the California Committee for the Protection of Human Subjects (2018-216-UCLA), and CMS (Data Use Agreement #52277). The analytic data sets were created using SAS 9.4 (Cary, NC) and analyses were performed using STATA 17 (College Station, PA).

## RESULTS

Between 2011 and 2016, 1,112,834 unique NH residents had 9,014,702 segments of time containing a POLST (Table 1). Overall, residents had a signed POLST containing orders for CPR with Full treatment 29.6% of the time and a signed POLST containing a DNR order with Selective treatment or Comfort care 27.1% of the time. Unsigned POLSTs accounted for 11.3% of residents’ time in the NH and residents had no POLST 28.2% of the time.

**Table 1:** Distribution of Cohort Characteristics, California Nursing Home Residents Enrolled in Fee-For-Service Medicare, 2011 to 2016^1^

	Overall^2^	Long-stay^2^	Short-stay^2^
Unique individuals, *N*	1,112,834	266,178	919,609
Number of segments, *N*	9,014,702	4,346,702	4,668,000
Segment duration, mean days	34.7	44.2	25.8
Gender—male (%)^1^	39.1	40.2	38.4
Age (%)^1^			
65–74	19.2	17.5	21.9
75–84	27.6	25.8	30.6
85+	33.6	36.0	29.7
Race/ethnicity (%)^1^			
Non-Hispanic White	61.2	56.9	68.0
Non-Hispanic Black	9.9	11.6	7.0
Hispanic	15.5	17.0	13.1
Asian	9.4	10.9	7.1
Other/unknown	4.0	3.5	4.8
Cognitive Function Scale (%)^1^			
Intact	45.6	33.1	66.2
Mild impairment	19.7	20.2	18.9
Moderate impairment	24.5	32.0	12.2
Severe impairment	10.2	14.8	2.7
Activities of Daily Living Scale (%)^1^			
Intact	11.0	10.1	11.6
Mild impairment	20.7	29.1	15.5
Moderate impairment	46.8	53.0	42.9
Severe impairment	21.5	7.8	30.0
Charlson Comorbidity Count (%)^1^			
0	8.1	1.5	18.6
1	5.8	5.5	63.9
2	12.2	12.1	12.4
3+	73.9	81.0	5.1
POLST intensity of care preferences (%)^1^			
CPR full, signed	29.6	28.0	32.2
CPR selective, signed	2.2	2.2	2.2
DNR full, signed	1.6	1.7	1.4
DNR selective, signed	15.5	17.9	11.5
DNR comfort, signed	11.6	14.4	7.1
CPR full, unsigned	6.8	5.2	9.2
CPR selective, unsigned	0.5	0.4	0.7
DNR full, unsigned	0.4	0.4	0.5
DNR selective, unsigned	1.9	1.8	2.2
DNR comfort, unsigned	1.7	1.6	1.8
No POLST (%)^1, 3^	28.2	26.4	31.2

^1^Results weighted for segment duration to reflect time in the nursing home

^2^Individuals can be classified as both a short-stay and long-stay nursing home resident in a calendar year

^3^Includes a POLST that has no Section A filled in

Among long-stay residents, POLST use and content were similar to the overall sample. Short-stay residents had a different distribution of POLST preferences compared to long-stay residents; they were less likely to have a signed POLST indicating a preference for DNR with Comfort care (7.1% vs. 14.4%) or DNR with Selective care (11.5% vs. 17.9%). Short-stay residents were more likely to have both a signed and unsigned POLST indicating CPR with Full care than long-stay residents (32.2% vs. 28.0% and 9.2% vs. 5.2%, respectively). Finally, short-stay residents were more likely than long-stay residents to have no POLST (or no Section A): 31.2% versus 26.4%, respectively.

In unadjusted annualized utilization results (Table 2), the overall mean number of hospitalizations was 14 per 100 resident-years, the mean number of days in the hospital was 120 per 100 resident-years, and the mean number of ICU days was 37 per 100 resident-years. Long-stay residents had a lower mean number of hospitalizations (4.9 per 100 resident-years), days in the hospital (67 per 100 resident-years), and ICU days (18 per 100 resident-years), compared to the overall sample. Short-stay residents had a higher mean number of hospitalizations (29 per 100 resident-years), days in the hospital (204 per 100 resident-years), and ICU days (67 per 100 resident-years) than the overall sample. Within each group, there is a gradient of effect across POLST treatment preferences with higher mean utilization associated with greater intensity of care preferences, ranging from CPR and Full treatment to DNR and Comfort-focused treatment. For example, among residents with signed POLST forms, those containing directions for CPR and Full treatment had 18 hospitalizations per 100 resident-years, DNR Full treatment 10 hospitalizations per 100 resident-years, and DNR Comfort care 5 hospitalizations per 100 resident-years.

**Table 2:** Unadjusted Annualized Hospital Utilization by POLST Use and Content, California Nursing Home Residents Enrolled in Fee-For-Service Medicare, 2011 to 2016^1,2^

	Number of hospitalizations^1, 2^			Days in the hospital^1, 2^			ICU days in the hospital^1, 2^		
	Overall	Long-stay	Short-stay	Overall	Long-stay	Short-stay	Overall	Long-stay	Short-stay
Overall average	14.1	4.9	28.7	119.6	67.0	203.5	36.8	17.9	67.1
POLST content									
CPR full, signed	17.7	7.4	32.0	151.9	95.4	230.5	48.1	27.5	76.8
CPR selective, signed	13.5	4.7	27.6	93.4	41.7	175.9	30.5	11.9	60.1
DNR full, signed	10.1	3.1	24.3	82.7	49.8	149.0	23.1	11.3	46.9
DNR selective, signed	8.2	2.1	23.4	52.4	20.1	132.7	15.1	4.9	40.4
DNR comfort, signed	5.3	1.6	17.3	37.0	16.5	103.9	10.6	4.0	32.0
Unsigned POLST content									
CPR full, unsigned	22.7	10.8	33.5	206.1	150.8	255.8	64.0	37.8	87.5
CPR selective, unsigned	17.4	6.8	26.2	136.3	82.2	181.3	42.3	22.3	58.9
DNR full, unsigned	13.5	4.1	24.2	96.1	47.1	152.5	28.7	17.0	42.3
DNR selective, unsigned	11.7	3.4	22.1	84.8	42.2	139.0	26.2	10.0	46.8
DNR comfort, unsigned	8.5	2.5	16.9	57.3	29.1	97.3	16.8	6.9	30.9
No POLST*	15.7	5.2	29.9	145.9	87.1	225.4	44.9	22.3	74.5

^1^Results weighted for segment duration to reflect time in the nursing home

^2^Per 100 person-years

*Includes POLST forms without Section A completed

In every category of intensity of care preference, overall utilization in all three measures was higher among residents with unsigned POLST forms compared to residents with signed POLST forms. This was nearly always the case for long-stay residents, but less consistently true for short-stay residents for whom utilization differed little between signed and unsigned POLST forms.

Multivariate results accounting for demographics, case-mix severity, and NH fixed effects demonstrated that overall, residents with a POLST containing an order for CPR and Full treatment had hospital utilization identical to residents with no POLST (Table 3). Across all NH residents, a gradient of decreased utilization was seen related to lower intensity of care orders from CPR Selective treatment with 1.7 fewer hospitalizations per 100 resident-years, DNR Full treatment 2.5, DNR Selective treatment 3.3 fewer, and DNR Comfort care 4.6 fewer hospitalizations per 100 resident-years compared to CPR Full treatment. The same pattern of utilization related to care intensity preferences was seen concerning hospital days and ICU days. Compared to residents without a POLST, residents with a POLST containing DNR Comfort care spent 56 fewer days in the hospital per 100 resident-years with 47 days and 41 days per 100 resident-years for residents with POLST forms containing orders for DNR Selective treatment and DNR Full treatment, respectively. Concerning days in the ICU, any care intensity less than CPR Full treatment resulted in fewer ICU days per resident-year and all were statistically significantly less than No POLST, but there was little difference across the gradient from CPR Selective treatment to DNR Comfort care.

**Table 3:** Adjusted Annualized Hospital Utilization by POLST Use and Content, California Nursing Home Residents Enrolled in Fee-For-Service Medicare, 2011 to 2016 ^1, 2, 3^

	Number of hospitalizations^1, 2, 3^			Days in the hospital^1, 2, 3^			ICU days in the hospital^1, 2, 3^		
	Overall	Long-stay	Short-stay	Overall	Long-stay	Short-stay	Overall	Long-stay	Short-stay
POLST content									
CPR full, signed	0.0	0.6	0.3	−3.3	5.0	7.9	−2.2	0.0	4.0
CPR selective, signed	−1.7	−0.2	−1.9	−40.8*	−30.2	−35.3	−14.7*	−11.4	−9.6
DNR full, signed	−2.5*	−1.0	−2.7	−41.1	−18.3	−72.3	−20.3*	−13.1*	−23.9
DNR selective, signed	−3.3*	−1.2*	−3.7*	−46.6*	−32.5*	−36.5*	−17.9*	−14.1*	−13.6
DNR comfort, signed	−4.6*	−1.9*	−6.7*	−56.0*	−42.5*	−52.4*	−21.7*	−16.5*	−20.6*
Unsigned POLST content									
CPR full, unsigned	−2.2*	−1.6	−3.3*	−25.3	−18.1	−26.6	−9.6	−6.9	−9.4
CPR selective, unsigned	−2.4	−0.6	−3.9	−30.8	−27.5	−34.1	−17.5	−16.7	−19.5
DNR full, unsigned	−3.0	−2.4	−4.3	−55.2	−57.8	−64.9	−18.3	−11.6	−34.9
DNR selective, unsigned	−2.5*	−0.6	−4.8*	−29.7*	−18.5	−27.5	−10.9*	−9.5	−10.2
DNR comfort, unsigned	−3.9*	−1.8*	−6.6*	−51.4*	−33.6*	−55.2*	−16.2*	−11.6	−21.7*

^1^Results weighted for segment duration to reflect time in the nursing home

^2^Per 100 person-years

^3^Models are adjusted for patient fixed effect, nursing home effect, male gender, age, race/ethnicity, Charlson Comorbidity Index, Cognitive Function Scale, Activities of Daily Living Scale, and imputation flags. Reference category is No POLST (including POLST with no Section A)

Number of segments is 8,867,843 overall, 4,322,880 for long-stay residents and 4,542,418 for short-stay residents. Number of unique residents is 1,112,834 (long-stay 266,178 and short-stay 919,609)

*p < 0.001

The pattern of utilization related to POLST orders in adjusted analyses was similar for long-stay and short-stay residents, but the magnitude of effect was consistently greater in the short-stay cohort across all utilization outcomes. Among short-stay residents, DNR Comfort care orders were associated with 6.7 fewer hospitalizations, 52 fewer hospital days, and 21 fewer ICU days (all per 100 resident-years) compared to short-stay residents without a POLST. Long-stay residents with a POLST containing orders for DNR Comfort care had 1.9 fewer hospitalizations, 43 fewer hospital days, and 17 fewer ICU days (all per 100 resident-years) compared to long-stay residents with no POLST.

A weaker and less consistent relationship between utilization and POLST content was seen in adjusted analyses for unsigned POLST forms across all utilization outcomes. The gradient of decreasing intensity of care from CPR Selective treatment to DNR Comfort care was smaller and less likely to be significantly different from No POLST compared to signed POLST forms. Concerning number of hospitalizations, an unsigned POLST containing CPR Full treatment—unlike a signed version—was associated with less hospitalization than having no POLST, but there was not a statistically significant relationship with hospital or ICU days. The overall findings concerning unsigned POLST forms largely reflect the findings among long-stay residents, whereas for short-stay residents, there was little difference in utilization between residents with signed and unsigned POLST forms.

## DISCUSSION

Among California NH residents, there is a direct relationship between intensity of care preferences in POLST and acute care utilization. NH residents with less intense treatment orders were hospitalized less often, and spent fewer days in the hospital and ICU. This is consistent with prior studies showing that treatment follows POLST content and demonstrates that across this chronically ill population, POLST is a reliable mechanism of translating care decisions into medical treatment across treatment settings.^3–5,12^ This effect is even stronger among short-stay residents. This has implications for the implementation of POLST: it is critical to make certain the POLST content reflects considered values in the context of prognosis and that POLST forms are completed when informed values dictate less aggressive treatment in the setting of clinical deterioration.

Unless otherwise noted, patients without known care preferences receive the highest intensity of care. These findings have implications for the 28% of NH resident-time spent without a POLST. If these residents had no POLST because they desired fully aggressive treatment, then they would have received the treatment desired in the setting of a medical emergency. This is evidenced by the identical utilization between residents with No POLST and those with a POLST indicating CPR Full treatment. If, however, these residents did not have a POLST completed because of inattention to advance care planning or communication obstacles,^13^ then utilization will not match goals and prognosis. Indeed, cognitively impaired residents during this period were *less* likely to have a POLST completed than those with intact cognition^14^ and cognitive and functional impairment are associated with preferences for less aggressive treatment.^15,16^ Furthermore, a study comparing NH residents with and without POLST forms found that residents without a POLST were less likely to have orders reflecting their elicited preferences.^17^

Orders in POLST forms without signatures have a weak relationship with clinical utilization. Discussions may be incomplete or decisions unsettled. Emergency medical personnel may not know whether to honor the forms. Utilization was higher if a POLST had a missing signature compared to a valid form. While only 11% of resident time was spent with unsigned forms, it behooves NH to reduce this. POLST content is more strongly associated with utilization for short-stay than long-stay residents. Although preference discussions may be more difficult with short-stay residents,^18^ these findings emphasize the importance of advance care planning with this group. If not carried out in the outpatient or hospital setting, it is incumbent on the NH clinician to engage the topic.

## LIMITATIONS

This study may not generalize to other time periods and geographic areas. California MDS Section S has not been validated and captures changes in POLST asynchronously rather than when they occur. POLST date is not collected, limiting causal inferences. The adjusted differences in magnitude of effect of POLST preferences between short- and long-stay residents may reflect difficulty in adjusting between these two groups. Long-stay residents are more homogeneous, older, sicker, and more impaired. Short-stay patients are more heterogeneous and it is likely that POLST preferences are more strongly associated with unmeasured severity of illness in this population, despite attempts to mitigate these effects analytically. It is not possible to fully account for this heterogeneity, especially in the short-stay population. Nevertheless, the size of the sample, mandatory nature of reporting, and methodologic approach support the robustness of the results.

## CONCLUSION

Among California NH residents, there is a direct relationship between intensity of care preferences in POLST and hospital utilization. These findings emphasize the importance of POLST capturing informed preferences based on understanding of prognosis. Furthermore, they emphasize the importance of capturing this information across all NH residents who desire less than fully aggressive treatment.

## References

[1] Morris JN, Fries BE, Morris SA (1999). *Scaling ADLs within the MDS*. J Gerontol A Biol Sci Med Sci.

[2] Tark A (2021). *Are We Getting What We Really Want? A Systematic Review of Concordance Between Physician Orders for Life-Sustaining Treatment (POLST) Documentation and Subsequent Care Delivered at End-of-Life*. Am J Hosp Palliat Care.

[3] Vranas KC (2021). *The influence of POLST on treatment intensity at the end of life: A systematic review*. J Am Geriatr Soc.

[4] Hickman SE (2010). *A comparison of methods to communicate treatment preferences in nursing facilities: traditional practices versus the physician orders for life-sustaining treatment program*. J Am Geriatr Soc.

[5] Lum H (2017). *Use of Medical Orders for Scope of Treatment for Heart Failure Patients During Postacute Care in Skilled Nursing Facilities*. J Am Med Dir Assoc.

[6] Reinhardt JP (2017). *End-of-Life Conversations and Hospice Placement: Association with Less Aggressive Care Desired in the Nursing Home*. J Soc Work End Life Palliat Care.

[7] Coalition for Compassionate Care, California Physician Orders for Life Sustaining Treatment. 2017.

[8] **Zingmond, D.S., et al.**, Changes over time in POLST use and content by race and ethnicity among California nursing home residents*.* J Am Geriatr Soc, 2023. 10.1111/jgs.18374 PMC10524124 37092747

[9] Thomas KS (2017). *The Minimum Data Set 3.0 Cognitive Function Scale*. Med Care.

[10] Charlson ME (1987). *A new method of classifying prognostic comorbidity in longitudinal studies: development and validation*. J Chronic Dis.

[11] CMS, Quality of Resident Care 2021. 2021.

[12] Hickman SE (2011). *The consistency between treatments provided to nursing facility residents and orders on the physician orders for life-sustaining treatment form*. J Am Geriatr Soc.

[13] **Heim Smith, N.L., et al.**, Reasons for Discordance Between Life-Sustaining Treatment Preferences and Medical Orders in Nursing Facilities Without POLST*.* Am J Hosp Palliat Care, 2022: p. 10499091221127996. 10.1177/10499091221127996 PMC10321076 36154692

[14] Jennings LA (2022). *Care preferences in physician orders for life sustaining treatment in California nursing homes*. J Am Geriatr Soc.

[15] Mazur DJ (1997). *How older patient preferences are influenced by consideration of future health outcomes*. J Am Geriatr Soc.

[16] Tsevat J (1995). *Health values of the seriously ill. SUPPORT investigators*. Ann Intern Med.

[17] Hickman SE (2021). *Do Life-sustaining Treatment Orders Match Patient and Surrogate Preferences? The Role of POLST*. J Gen Intern Med.

[18] Lam K, Haddock L, Yukawa M (2022). *More POLST forms are being completed in nursing homes, but is this meaningful?*. J Am Geriatr Soc.

